# Neonatal Appendicitis (Part 1): A Review of 52 cases with Abdominal Manifestation

**Published:** 2015-01-10

**Authors:** V Raveenthiran

**Affiliations:** M.Ch, FRCS (Glas)Department of Pediatric Surgery, Sri Ramasamy Memorial (SRM) Medical College SRM University, Chennai 603203, India

*“No age is free from risk of an attack Of inflammation in this cul-de-sac” - Zeta (Cope Z) Acute abdomen in rhymes*

Although no age is free from the risk of appendicitis, it is extremely uncommon in newborns. Credit of describing the first neonatal case is disputed between Diess (1908) [1] and Albrecht (1905).[2] Although Lillenthal reported a survivor of scrotal appendicitis in 1908, [2] 45 years elapsed before Meigher and Lucas [1] in 1952 documented the first survivor of abdominal disease. Another 35 years passed before proving as to what Sir Zachery Cope remarked as “No age” really extends into prenatal period. Martin - Glen (1986) [3] and Narasimharao et al. (1987) [4] recorded antenatal appendicitis. A further 15 years went before hearing about the first successful laparoscopic appendicectomy in neonate by Efrati et al. [5] Intrigued by the tardy progress, Athena looked for a review article to dwell more on this subject. Although Karaman et.al [2] summarized 141 cases collected over a century (1901 - 2000), Athena is disappointed that the entity has largely remained anecdotal and almost all the published reports are individual case reports or at the best a small series. Therefore, she decided to critically analyze the published data to solve certain unanswered questions. 

**Unverified Assumptions**

Hundred years ago, Wangensteen proved that appendicitis is caused by luminal obstruction. Recumbence of newborn and funnel shape of fetal appendix are said to protect against appendicular blockage. Milk, unlike adult food, does not leave behind undigested residue or fecal pellets. Newborns are least likely to be exposed to infective agents such as adenoviruses, which are known to cause lymphoid hyperplasia. Thus, many of the risk factors operating in grown-ups are absent in newborns, which is why they are least prone for appendicitis. Three etiological hypotheses abound to explain the rare occurrence of neonatal disease. Martin and Perrin [6] suggested that obstruction caused by Hirschsprung disease could play a role in the pathogenesis. Bax et al. [7] proposed that neonatal appendicitis (NA) is actually a limited form of necrotizing enterocolitis (NEC). The observation that more than 50% of infants with appendicitis are preterm [2] adds strength to Bax’s theory because 90% of NEC is also found in premature babies. Wangensteenian surgeons contemplate etiological role of obstruction due to pellet like stool of cystic fibrosis and meconium ileus. But none of the three theories are scientifically proved. 

Mortality of NA was as high as 78% between 1901 and 1975. It rapidly declined to 33% during 1976-84 owing to rapid advances in antibiotic therapy, neonatal intensive care and diagnostic modalities. Further drop in death rate to 28% during 1985-2003 was only a modest improvement. [2] These figures cause great concern in the era when corresponding mortality in grown-ups is approaching zero per cent. Several factors are incriminated for this high fatality. They include (1) diagnostic and therapeutic delay due to lack of specific clinical features and rarity of the disease; (2) early perforation due to fragility of neonatal appendix; (3) poorly walled off infection due to underdeveloped omentum; (4) immature immune system and (5) limited physiological reserve of preterm babies. All these assumptions have not been subjected to rigorous scientific testing.

**Unanswered questions**

 Foregoing hypothetical assumptions made Athena inquisitive of the following concerns:

1. Is there a causal relationship between NA and Hirschsprung’s disease?
2. How often is cystic fibrosis or meconium ileus associated with NA?
3. Is NA a form of NEC? How often are the risk factors of NEC seen in NA?
4. What is the current mortality of NA? Is it unusually high?
5. Is the mortality affected by gestational maturity, diagnostic delay or perforation?
6. Does the perforation occur early and is there a correlation between it and therapeutic delay?
7. How often the infection or perforation is effectively walled off?
8. Is there a clue for early diagnosis?
9. Which is the most useful investigation?
10. Is a particular group of neonates more vulnerable to NA than others?

**Athena’s Plot**

Athena is not for mixing apples and oranges. Clubbing the current literature with that of remote past is meaningless because of the significant advancements made recently. Therefore, Athena restricted her analysis to reports published during the last 25 years (1990 - 2014). Inflammed appendix in hernial sac (Amyand hernia) has a totally different outlook from that of the abdominal disease. Hence, Athena excluded Amyand hernia and intends to examine it separately. She searched Pubmed, Google Scholar, Embase, Indmed and AJOL using a keyword combination of ‘neonate’, ‘newborn’ and ‘appendix’. She excluded 5 neonates reported in non-English articles,[8-11] 2 patients who were actually treated prior to 1990 [12] and one [13] of the duplicate publications. [2, 13] Definitions of neonatology terms were similar to that of WHO convention. However, infants who had disease onset during neonatal period but presented later than 28 days were also included. Athena could collect 52 cases of neonatal appendicitis treated and reported between 1990 January and 2014 December. [1,2,5,12,14-50]

**Geographic Distribution**

Athena found that maximum number of cases have been reported from India (n=10), which is followed by Turkey (n=8), USA (n=5), UK (n=4), and Canada (n=4). The high incidence in India does not appear to be due to her global first-rank in preterm population. [51] This impression is supported by the fact that only one case has been described from China, [31] which is in the second position of preterm census. Further there are no reports from Nigeria, Indonesia, Malawi and Congo, which are in successive position of having the highest preterm birth rate. Therefore, Athena concludes the skewed distribution could be a phenomenon of publication bias or genetic susceptibility.

**Sex Ratio**

Karaman et al. [2] showed a clear male preponderance with a male: female ratio of 3:1. But Athena finds a narrowing gap in sex ratio with 56% in males and 40% in females. (Table 1) It is not clear if the change is due to selection bias or due to an alteration in the disease pattern. The latter appears to be plausible because Karaman et al had also shown a similar trend. While analyzing the data of 3 time-intervals namely 1909-75, 1976-84 and 1985-2003, they calculated male: female proportion as 60:27, 60:40 and 50:36 respectively. 

**Gestational Maturity**

 Traditionally, preterm neonates are considered to be more vulnerable for appendicitis. Nearly 52% were premature babies in Karaman’s review [2]. But Athena finds term infants being more affected than preterm (48% vs. 37%). This is corroborated by the fact that an equal proportion of the neonates were having optimal and suboptimal birth weight. (Table 1) These observations strengthen the hypothesis that the disease pattern is changing over the time. 

**Figure:**
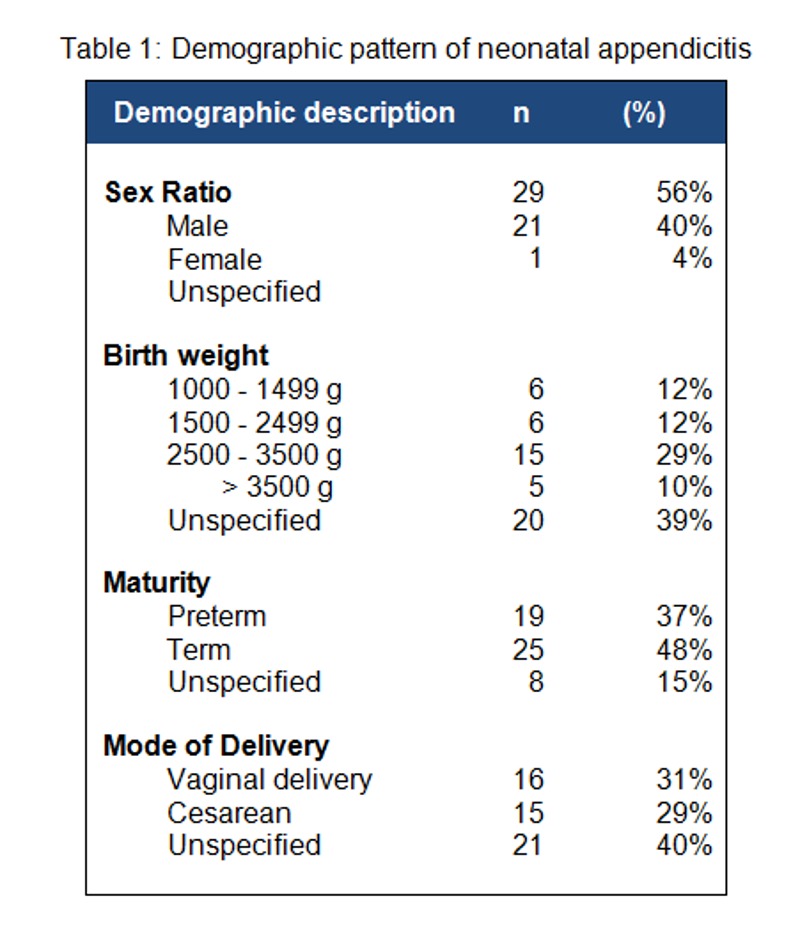
Table 1: Demographic pattern of neonatal appendicitis

**Etiological role of Co-morbidity and Risk factors**

 Athena could not find a single report of cystic fibrosis or meconium ileus in NA. Even Karaman, [2] over a period of 100 years, could find only one case of cystic fibrosis in 128 NA. Therefore, the alleged correlation between the two pathologies appears to a medical myth. 

Athena’s series had only 4 cases of Hirschsprung disease co-existing with inflammed appendix. None of them had features of enterocolitis of megacolon. Prior to 1990, this combination has been reported in only 9 neonates [44] but not in older children. Sarioglu et al. [44] over a period of 18 years, picked up 2 NA among 302 neonates with Hirschsprung’s disease. Extreme rarity of the association precludes any meaningful analysis. Periappendicitis without transmural infiltration of neutrophils is said to be characteristic of appendicular perforation due to congenital megacolon. [6] This could not be verified because fine details of appendicular histology are not usually included in published case reports. For these reasons, coincidence of colonic aganglionosis and NA will remain a clinical curiosity rather than a scientific fact.

Generalized peritonitis and intestinal congestion of perforated appendicitis is difficult to distinguish from that of NEC. Athena noted that one or more risk factors of NEC were present in 23 (44%) neonates suffering from appendicitis. Peri-natal or pre-morbid asphyxia was reported in as many as 17 (33%) instances. Prolonged rupture of membrane, chorio-amnionitis and maternal sepsis were present in 7 (13%) cases. Serious congenital heart disease (n=3) and maternal smoking (n=1) were also noted. Therefore, the claim of NA being a form of NEC has some substance and it deserves further investigation. A well designed animal study is indicated to know as to why NEC changes are confined to the appendix. 

**Clinical Diagnosis**

 Definitive diagnosis was made clinically in 3 neonates and at autopsy in 2 infants. All others were diagnosed retrospectively after surgical exploration. Diagnostic role of laparoscopy seems to be underutilized because only 5 cases have been described so far. [5,20,34,42] Abdominal distension (89%), vomiting (54%), abdominal tenderness (48%), restlessness or lethargy (36%) and fever (31%) were the most common symptoms. [Table 2] Admittedly, they are non-specific and hence may not narrow down the clinical diagnosis. Contrary to general belief 18 of the neonates (35%) had one or more localizing signs. Interestingly only 3 of them (17%) were correctly diagnosed prior to laparotomy. The high rate of misdiagnosis despite the presence of localizing sign attests the old proverb, “Eyes can’t see what the mind does not know”. Signs of perforated appendix such as flank erythema or edema, palpable mass and tenderness are also seen in NEC. Understandably, the most common misdiagnosis was NEC in 32% cases. Nevertheless on careful analysis, Athena found that these signs when occurring exclusively in RIF indicate appendicitis rather than NEC. 

**Figure:**
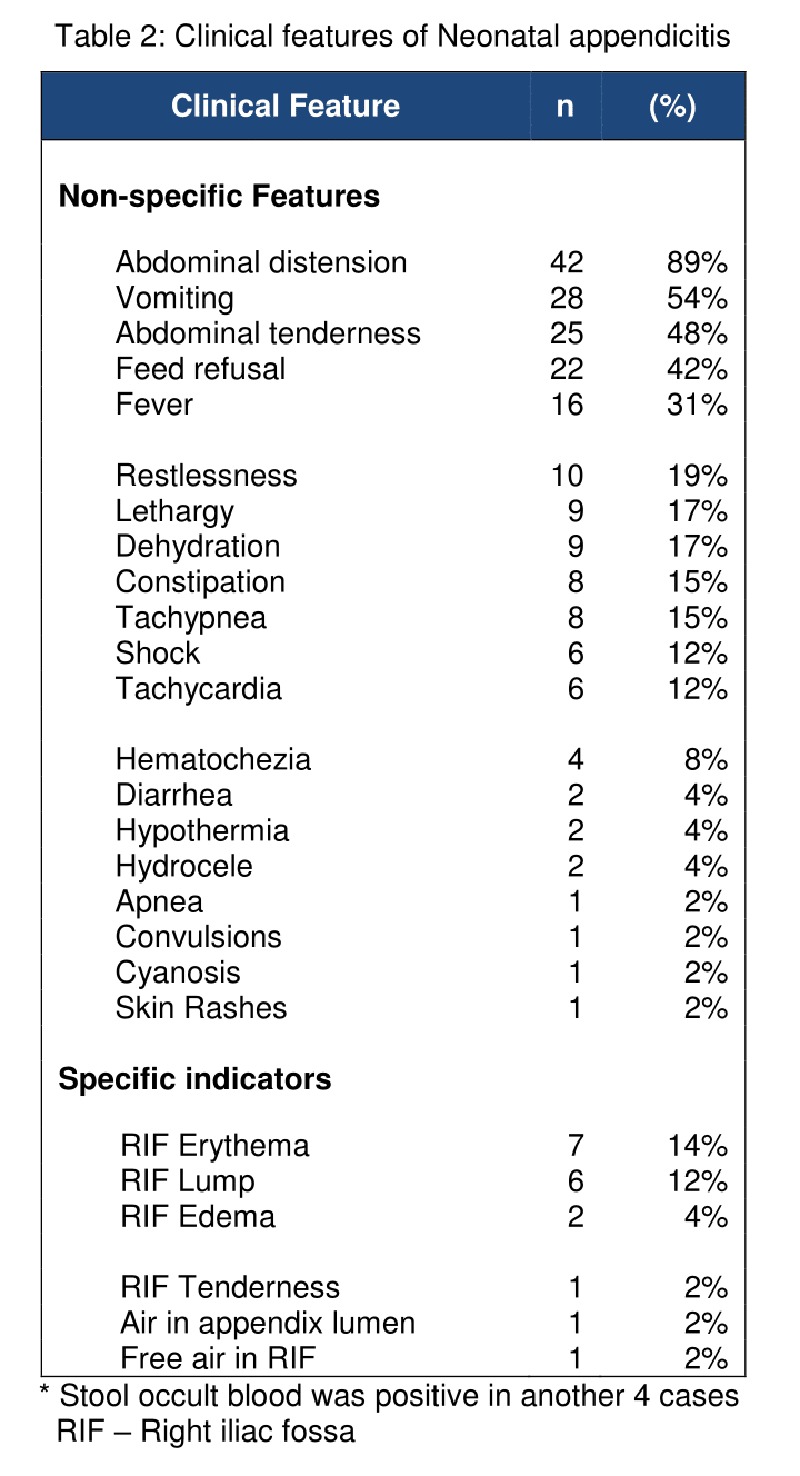
Table 2: Clinical features of Neonatal appendicitis

**Most useful Investigation**

 Leukocyte count and ultrasonography (USG) showed poor yield. Free fluid or mass in RIF (8%) demonstrated by USG are useful but not conclusive of appendicitis. On the other hand, USG distracted the clinical attention towards coincidental cystic lesions. Actually, a few infants had undergone laparotomy for suspected hydronephrosis [14] or duplication cyst [39,42] based on the USG reporting and the correct diagnosis turned out to be an intra-operative surprise. Sepsis screening such as C-reactive protein and blood culture were positive in only 27% of cases. 

 Pneumoperitoneum is the single most useful sign which was seen in 23 of the 44 (52%) patients with perforation. Correct pre-operation diagnosis was possible in 2 instances when the free gas was seen in RIF or inside the appendicular lumen. Plain radiographs, although not diagnostic of appendicitis, are helpful in detecting complications. 

**Perforation and Walling off Infection**

 Approximately 85% of the infants had appendicular perforation at laparotomy. The mean delay between the onset of symptoms and definitive therapy was 8+3.5 days in uncomplicated cases and 3.3+3 days in perforated appendices. Using Student’s t-test, Athena found the difference be statistically significant (P = 0.044). Usually perforation is expected to be more common when there is inordinate delay. Therefore, the paradox of shorter delay in perforation group necessitated further dissection of data by compartmentalizing pre-hospital procrastination and post-admission delay. The mean delay between admission to NICU for abdominal symptoms and definitive treatment was also significantly longer in uncomplicated group (5.3 + 3.2 days) than in perforation group (1.8 + 2.3 days). However, the mean duration of pre-hospital symptoms did not differ significantly between the two groups (1.7 + 2.3 vs 2.7 + 0.6 days in perforated and non-perforated groups respectively). From this analysis Athena infers that diagnostic delay did not increase the perforation rate. Conversely, neonates with appendicular perforation were diagnosed and treated earlier than those without it. Even in the absence of correct clinical diagnosis, perforation - as indicated by pneumoperitoneum - appears to have prompted surgical exploration. As a note of caution, Athena is also aware that the statistics may be deceptively fallacious due to extremely small sample size.

 The present analysis confirms the traditional view that infection of perforated appendix is poorly walled off in newborn. Appendicular abscess and mass formation were noted in only 9 (17%) infants. However, this account may be misleading because in 64% of published reports a specific description of operation finding is missing. 

**Mortality**

 Athena computed a disturbingly high mortality of 23 per cent even in this modern era. The death rate was not affected by sex of the infant, birth weight, gestational maturity, mode of delivery or any of the individual symptoms. One would expect mortality of perforated appendicitis be greater than that of uncomplicated cases. Perplexingly, only 8 out of 44 neonates (18%) with perforation died while 4 out of 7 (57%) without perforation expired. Using 2-tailed Fisher’s exact test, Athena found the difference be statistically significant (P = 0.045). The paradox is easy to explain by correlating the fact that neonates with perforated appendicitis underwent early surgical intervention. Benefits of early appendicectomy appear to offset the adversities of perforation. 

## Conclusion

To conclude, NA does not appear to have any causal relationship with Hirschsprung’s disease or cystic fibrosis. Association between NA and NEC deserves further investigations because both share the same spectrum of risk factors. Clinical diagnosis of NA is possible if abdominal wall edema, erythema, palpable mass or tenderness is noted exclusive in RIF. USG is often misleading and unhelpful. Pneumoperitoneum noted in radiographs, despite failing to clinch the correct diagnosis, is useful in identifying perforation and in prompting early surgical intervention. There appears to be no correlation between perforation and diagnostic delay. The current death rate of 23% is unacceptably high. The inverse correlation of mortality with perforation rate could be due to therapeutic advantage of early intervention in complicated cases. 

**Epilogue**

 The real incidence of NA is not known. Athena chanced to see an abstract by Oyetunji et al. [52] wherein the authors have analyzed epidemiological characteristic of NA using a national database. It is a great loss to medical science that the authors did not pursue to publish the full-text of it.

Recently Bengtsson and Houten [53] introduced a new term neonatal appendicopathy which includes primary-, secondary- and peri appendicitis. Although Athena concurs with the authors on the practical difficulties of distinguishing true appendicitis from inflammation of appendix secondary to NEC, she is reluctant to buy the authors’ arguments and the proposed terminologies. For example, periappendicitis is in fact secondary appendicitis. Inflamed appendix in hernia sac is a unique clinical presentation rather than secondary appendicitis.

## Footnotes

**Source of Support:** Nil

**Conflict of Interest:** The author is Editor of the journal. But he did not take part in the evaluation or decision making of this manuscript. The manuscript has been independently handled by two other editors.

